# Pain Phenotypes in Knee Osteoarthritis: Between Mechanistic Taxonomy and Clinical Complexity

**DOI:** 10.1111/os.70217

**Published:** 2025-12-03

**Authors:** Antonio Alcántara Montero

**Affiliations:** ^1^ Centro de Salud Trujillo Cáceres Spain

Knee osteoarthritis (KOA) is a chronic and highly prevalent condition that ranks among the leading causes of disability in older adults worldwide [1]. Traditionally, it has been approached from a structural perspective—focused on cartilage deterioration, subchondral bone damage, and synovial inflammation. However, recent years have seen the emergence of a more sophisticated understanding of pain as a multidimensional phenomenon, which often fails to correlate directly with the anatomical damage observed [2].

This paradigm shift has led to the development of mechanistic models for classifying pain in KOA, aiming to improve diagnostic and therapeutic precision. In this context, the article by He et al. [3] proposes a taxonomy based on the criteria of the International Association for the Study of Pain (IASP), distinguishing between nociceptive, neuropathic, and nociplastic pain. Furthermore, it subdivides nociceptive pain into three categories: inflammatory, mechanical, and pain related to bone marrow lesions. This classification allows each type of pain to be associated with specific therapeutic strategies, representing a step forward toward personalized medicine.

The proposal is clear, well‐structured, and clinically appealing. However, its practical application presents several challenges. First, accurately identifying the pain phenotype in each patient is not always straightforward. Although tools such as the PainDETECT questionnaire [4] and the Central Sensitization Inventory [5] exist, their systematic use in orthopedic practice is limited, and their sensitivity may be affected by factors such as educational level, comorbidities, or the patient's emotional context. Moreover, their application in orthopedic settings requires further cultural and clinical validation. Linguistic adaptation and assessment of diagnostic sensitivity in chronic musculoskeletal pain populations are essential to ensure their accuracy and relevance.

Second, dividing pain into discrete categories may be overly rigid. Numerous studies have shown that pain in KOA evolves over time, and patients may transition from predominantly nociceptive pain to neuropathic or nociplastic forms, especially in advanced stages or following surgical interventions [6, 7, 8]. This evolution is not always linear and may be influenced by factors such as persistent inflammation, altered central processing, or the presence of sleep and mood disorders [9].

In this regard, the concept of “mixed pain” has gained relevance as a useful clinical category to describe the simultaneous or sequential coexistence of diverse mechanisms. This concept has gained increasing recognition in recent years, particularly in orthopedic contexts such as osteoarthritis and post‐surgical pain. Its inclusion in clinical guidelines and multimodal intervention studies highlights the need for flexible therapeutic approaches that address both nociceptive and neuropathic mechanisms. Although not yet formally recognized by the IASP, its inclusion in clinical guidelines would allow for a more realistic and adaptive approach. In patients with mixed pain, the therapeutic strategy must be multimodal, combining pharmacological and non‐pharmacological interventions, and adapting to the evolution of pain and the individual response of each patient [10].

Another aspect that deserves attention is the role of nociplastic pain, which is often underdiagnosed in orthopedic settings. This type of pain, characterized by central amplification without evident tissue damage, may explain the persistence of pain in patients with mild KOA or even after technically successful arthroplasty [8]. Its management requires a paradigm shift, including pain education, tailored physical exercise, sleep hygiene, and cognitive‐behavioral therapy [9]. Although supported by evidence, these interventions remain underutilized in daily practice.

Moreover, He et al.'s proposal does not delve deeply into the interaction between mechanisms or the temporal evolution of pain. In clinical practice, KOA pain changes not only in intensity but also in quality, location, and response to treatment. This variability requires close clinical monitoring and therapeutic flexibility that goes beyond initial classification. Taxonomic rigidity, while useful for research and education, may limit clinical effectiveness if not accompanied by an integrative vision.

Finally, it is essential to emphasize that KOA pain cannot be understood without considering the patient's biopsychosocial context. Factors such as anxiety, depression, social isolation, or perceived control over the disease decisively influence the pain experience and treatment response [9]. Incorporating these elements into assessment and therapeutic design is essential to provide truly personalized care.

Altogether, the classification of pain phenotypes in KOA represents a necessary step to move beyond the traditional symptomatic approach. However, its implementation requires a profound transformation in clinical practice, including specific training, validation of diagnostic tools, and development of decision‐making algorithms that integrate pain evolution and patient experience. Recognizing pain mechanisms not only improves therapeutic efficacy but also humanizes treatment by adapting it to the patient's lived experience.

Ultimately, understanding pain in knee osteoarthritis from a mechanistic perspective not only allows for more precise treatment but also dignifies the patient's experience. The classification proposed by He et al. is a significant advance, but its clinical application demands flexibility, specific training, and an integrative approach that considers the temporal evolution of pain and its biopsychosocial dimension. Incorporating these approaches into daily practice is not merely a matter of therapeutic precision, but an ethical imperative for those who live with pain as part of their everyday lives.

## Author Contributions

Antonio Alcántara Montero conceptualized the manuscript, conducted the critical analysis of the referenced article, and drafted the full text. He performed the literature review, integrated the clinical and theoretical perspectives, and refined the final version of the manuscript. The author approved the submitted version and takes full responsibility for its content.

## Funding

The author has nothing to report.

## Ethics Statement

The author has nothing to report.

## Conflicts of Interest

The author declares no conflicts of interest.

## Data Availability

Data sharing not applicable to this article as no datasets were generated or analyzed during the current study.
